# Phenotypic Detection of Genitourinary Candidiasis among Sexually Transmitted Disease Clinic Attendees in Ladoke Akintola University Teaching Hospital, Osogbo, Nigeria

**DOI:** 10.1155/2015/401340

**Published:** 2015-05-07

**Authors:** Oluranti J. Obisesan, Olugbenga A. Olowe, Samuel S. Taiwo

**Affiliations:** ^1^Department of Medical Microbiology and Parasitology, Ladoke Akintola University of Technology Teaching Hospital, PMB 5000, Osogbo, Nigeria; ^2^Department of Medical Microbiology and Parasitology, College of Health Sciences, Ladoke Akintola University of Technology, PMB 4400, Osogbo, Nigeria

## Abstract

The management of genitourinary candidiasis (GC) is fraught with challenges, especially, in an era of increasing antifungal resistance. This descriptive cross-sectional study conducted between May 2013 and January 2014 determined the prevalence and characteristics of GC and the species of* Candida *among 369 attendees of a Sexually Transmitted Disease (STD) clinic of Ladoke Akintola University Teaching Hospital, Osogbo, Nigeria. Appropriate urogenital specimen collected from each attendee was examined by microscopy and culture for* Candida*, with preliminary species identification by CHROMAgar* Candida *and confirmation by Analytical Profile Index (API) 20C AUX. The age range of attendees was 1-80 years, mean age was 36.32 ± 11.34 years, and male to female ratio was 1 to 3. The prevalence of genitourinary candidiasis was 47.4%, with 4.9% in males and 42.5% in females (*p* < 0.0001). The age groups 31–45 and 16–30 have the highest prevalence of 23.3% and 16.8%, respectively. The species of* Candida *recovered include* Candida glabrata *46.9%,* Candida albicans *33.7%,* Candida dubliniensis *9.7%,* Candida tropicalis *5.7%,* Candida krusei *1.7%,* Candida lusitaniae *1.7%, and* Candida utilis *0.6%. This study reported non-*C. albicans *Candida, especially* C. glabrata, *as the most frequently isolated species in GC, contrary to previous studies in this environment and elsewhere.

## 1. Introduction

Genitourinary candidiasis is a fungal infection of the genital and urinary tracts caused by* Candida,* presenting as vulvovaginal candidiasis in women, balanitis and balanoprostitis in men, and candiduria in both sexes [1, 2].* Candida* species are normal microbiota of the respiratory tract, gastrointestinal tract, mouth, and vagina, and cause opportunistic infections when altered host conditions enable the fungus to proliferate. Although genitourinary candidiasis can occur in both immunocompetent and immunocompromised persons, it is a cause of morbidity and mortality in immunocompromised patients in particular [3]. Risk factors associated with genitourinary candidiasis include sexual contacts, poor genital hygiene, urinary instrumentation, urinary obstruction, and incomplete bladder emptying [4–6]. Among* Candida* species implicated, epidemiological studies have shown that* C. albicans* is the most commonly isolated species [6–9]. The newly emerging non-*Candida albicans* Candida, including* Candida glabrata, Candida krusei*,* Candida tropicalis, Candida lusitaniae,* and* Candida parapsilosis,* are however now implicated as causative agents [10–14] of genitourinary candidiasis.

The incidence of genitourinary candidiasis varies in relation to gender, host susceptibility, and hospital settings. The incidence is higher in women and among hospitalized patients [7–9], particularly in intensive care [7] and burn units [15]. The incidence is also high in pregnant women and in HIV-infected patients [16, 17], in patients with uncontrolled diabetes mellitus [18–21], and in individuals with multiple sexual partners [5, 16, 22].

Genitourinary candidiasis can be symptomatic or asymptomatic [23, 24]. Vulva pruritus and vaginal discharge are the most frequent symptoms of vulvovaginal candidiasis [25]. The discharge, which is usually adherent but not offensive, is typically cottage cheese-like in appearance but may range from watery to homogenously thick discharge. Other symptoms include vaginal soreness from scratching, vulva burning, dyspareunia, and dysuria. There may be erythema and swelling of the labia and vulva on examination but the cervix usually appears normal [25, 26]. Asymptomatic candiduria often occurs in hospitalized patients with indwelling catheters, and these patients usually show none of the signs or symptoms associated with urinary tract infection [3]. Although candiduria frequently occurs, especially in catheterized patients, symptomatic candida cystitis is rare, as bladder is relatively resistant to* Candida* invasion. When it occurs, however, it may present with signs and symptoms of bladder irritation including dysuria, hematuria, frequency, urgency, and suprapubic tenderness [27]. Conversely, symptomatic candida cystitis is rare in noncatheterized patients; however prostatic abscess caused by* Candida* species is not uncommon in male patients with diabetes mellitus [15, 28].

In our clinic, the diagnosis of genitourinary candidiasis is often made on clinical ground and by conventional laboratory detection of* Candida* with wet film/Gram smear microscopy, and culture on Sabouraud Dextrose Agar followed by identification based on colonial morphology, germ tube test, and formation of chlamydospores on cornmeal agar [27]. However other non-*C. albicans* species such as* C. glabrata* and* C. dubliniensis* are known to exhibit morphologic and biochemical characteristics indistinguishable from* C. albicans.* This conventional method therefore may not differentiate the* Candida* species, and this gives tendency to overreporting of* C. albicans* as the most frequent cause of genitourinary candidiasis.

In view of the changing epidemiology of fungi infections occasioned by worldwide increased incidence of immunocompromising diseases coupled with increased use of immunosuppressive therapies and resistance to traditional antifungi agents [29], it is imperative to employ methods that will identify species of* Candida* that may exhibit different susceptibility to currently available antifungi agents. These methods include the use of selective chromogenic media such as CHROMAgar which allow rapid identification of different* Candida* species based on colour changes produced from utilization of chromogenic substrates, facilitating detection of even mixed infections with more than one* Candida* species [29]. Commercial identification kits such as the Analytical Profile Index (API) that measures metabolic activities of* Candida,* ELISA antigen detection, and molecular methods such as the Polymerase Chain Reaction (PCR) tests can reliably differentiate* C. albicans* from non-*C. albicans* Candida species [11, 13]. However, only few laboratories in our environment have financial capability to employ these methods for routine identification of* Candida* isolates.

In this study, we employed conventional culture for initial isolation of* Candida* and identify the species of* Candida* using CHROMAgar* Candida* and then confirmed the species by the API 20C AUX. The objectives are to determine the prevalence and characteristics of genitourinary candidiasis as well as the species of* Candida* among attendees of our Sexually Transmitted Disease (STD) clinic. This information is necessary for proper management of genitourinary candidiasis in view of recent reports in our hospital of apparent failed treatment among women treated for vaginal candidiasis with azole antifungi agents.

## 2. Materials and Methods

### 2.1. Study Design and Setting

A descriptive cross-sectional study was conducted during the period May 2013 and January 2014 at Ladoke Akintola University Teaching Hospital, Osogbo, Nigeria. The research project proposal was approved by the Ethical Committee of the institution. Informed consent was obtained from each attendee prior to the start of the study.

### 2.2. Subjects

All consenting attendees (all age groups and gender) of the Sexually Transmitted Disease (STD) clinic for purposes of evaluation on account of STDs or genitourinary complaints were recruited into the study. No attendee was excluded except those who did not give informed consent. Sociodemographic and clinical data of each attendee were collected into an interview protocol form. Our STD clinic has recently been renamed Genitourinary Medicine (GUM) clinic to accommodate all cases of genitourinary complaints aside STDs.

### 2.3. Specimen Collection

Appropriate specimen types were collected in relation to gender, age, and complaints. Vulvovaginal and urethra swabs were collected by qualified medical personnel using cotton tipped swab. High vaginal and endocervical swabs were collected after speculum examination from posterior fornix and endocervix, respectively. All swabs were immediately transported to the Medical Microbiology Laboratory of the hospital for processing. For urine specimen, each adult attendee was instructed (verbal and written) to collect 10 mL of a clean-catch midstream urine specimen into a sterile screw-capped wide-mouth container and immediately deliver it to the laboratory. For children, parents or guardians were instructed appropriately how to collect urine specimen from the child into sterile bottle.

### 2.4. Wet Film Microscopy

A swab stick containing specimen was emulsified with small volume of normal saline (0.9% NaCl) on a clean grease-free glass slide and covered with a cover slip. The preparation was examined under the microscope using 10x and 40x objective lenses for oval budding yeast presumed to be* Candida* [27].

A deposit of centrifuged urine was placed on a glass slide, covered with a cover slip, and examined microscopically at 400x magnifications for presence of yeast cells and other cells such as trichomonads and clue cells.

### 2.5. Gram Stain Microscopy

Smears of swabs were made from the saline mixture and urine deposit on a clean glass slide. Each smear was air-dried and heat-fixed by passing over a Bunsen burner flame three times to fix the slide. The slide was Gram-stained using recommended procedure and then examined for oval yeast cells [27, 30].

### 2.6. Qualitative Culture and Identification of* Candida* Species

Swabs and urine specimens were inoculated onto Sabouraud Dextrose Agar (Oxoid, UK) supplemented with chloramphenicol and incubated at 37°C for 48 hours for qualitative culture analysis. Colonies from SDA were first Gram-stained and those that showed Gram positive yeast were then subcultured on CHROMAgar* Candida* (Paris, France) selective and differential medium. With the inclusion of chromogenic substrates in the medium, the colonies of* C. albicans*,* C. tropicalis*,* C. glabrata,* and* C. krusei* produce different colors, thus allowing the detection of these* Candida* species. Colonies of* C. albicans* appeared as light to medium green,* C. tropicalis* colonies as dark blue to metallic blue,* C. dubliniensis* as dark green,* C. krusei* colonies as pale pink with white edge rough,* C. glabrata* as pink to violet surface with pale edges,* C. lusitaniae* as pink to lavender, and* C. utilis* as glossy pink. All* Candida* species identified on CHROMAgar were further subjected to sugar assimilation and fermentation tests using API* Candida* (API BioMérieux, France) to confirm each species. Fungi susceptibility testing could not be done due to lack of facility in our laboratory at the time of this study.

### 2.7. Data Entry and Statistical Analysis

Sociodemographic, clinical, and microbiological data were entered into SPSS (Statistical Package for the Social Sciences) version 16.0 on a Window 7 laptop computer for analysis. The Chi-square or Fisher exact test was used to estimate significant differences between categorical variables. Values were considered to be statistically significant when the *p* value obtained was less than 0.05.

## 3. Results

### 3.1. Study Population

A total number of 369 attendees (mean age: 36.32 ± 11.34 years, age range: 1 to 80 years) were studied of which 91 (24.7%) were males and 278 (75.3%) were females, giving a M : F ratio of 1 : 3. The age group distribution and sociodemographic characteristics of the study population are shown in Table 1. The age groups 31–45 and 16–30 constitute the largest group patronizing the STD clinic with 53.9% (199 of 369) and 28.2% (104 of 369), respectively. Of the 369 attendees, 86 (23.3%) were pregnant women, 94 (25.5%) had underlying HIV infection, 70 (18.9%) had diabetes mellitus (DM), 33 (8.9%) had underlying urinary tract infection (UTI), and 86 (23.3%) were visiting the clinic for fear of having contacted STDs (venereophobia) but had no underlying medical condition or illness.

### 3.2. Prevalence and Characteristics of Genitourinary Candidiasis

**Figure 1:**
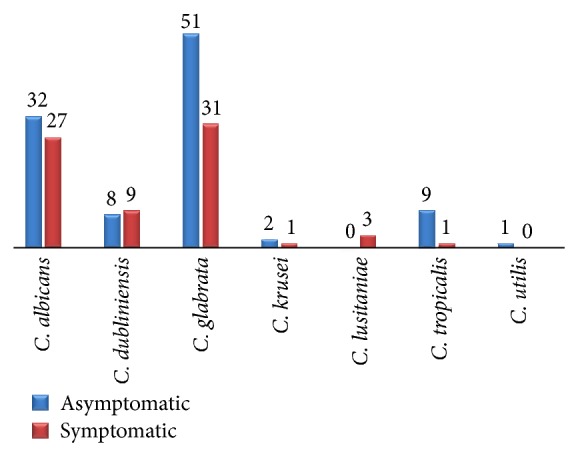
Distribution of* Candida* species in symptomatic and asymptomatic attendees.

Table 2 shows the prevalence of genitourinary candidiasis in the study population. Of the 369 attendees, 175 were positive for* Candida*, giving a prevalence of genitourinary candidiasis of 47.4% (175 of 369) in the study, with 4.9% (18 of 369) in males and 42.5% (157 of 369) in females (OR 1.9, *p* < 0.0001). The age groups 31–45 and 16–30 which constitute the largest groups patronizing the clinic also have the highest prevalence of genitourinary candidiasis with 23.3% and 16.8%, respectively (*p* = 0.0034). Symptoms of genitourinary candidiasis were seen in only 72 (41.1%) of the 175 attendees positive for* Candida* which include 39 pregnant women, 1 HIV-infected individual, 25 DM patients, 7 UTI patients, and none of the apparently healthy attendees. One hundred and three (58.9%) of those positive for* Candida* were asymptomatic and include 12 pregnant women, 39 HIV-infected individuals, 9 DM patients, 3 UTI patients, and 40 apparently healthy individuals (Table 3 and Figure 1).

### 3.3. Frequency of Isolation of* Candida* Species

Table 4 shows the species of* Candida* recovered in the study and their frequency of isolation. They include* Candida glabrata* 82 (46.9%),* Candida albicans* 59 (33.7%),* Candida dubliniensis* 17 (9.7%),* Candida tropicalis* 10 (5.7%),* Candida krusei* 3 (1.7%),* Candida lusitaniae* 3 (1.7%), and* Candida utilis* 1 (0.6%). Both* C. albicans* and non-*C. albicans* Candida species were recovered across all categories of attendees without any particular pattern or preference (*p* = 0.6455 > 0.05, Table 5). The recovery rate of* Candida* species from clinical specimens was highest with urine samples followed by vulvovaginal swabs and least from urethral swab (Table 6).

## 4. Discussion

The incidence of genitourinary candidiasis has dramatically increased in recent years as a result of increase in the incidence of immunodeficiency diseases such as HIV/AIDS, malignancies, metabolic dysfunctions, and the increasing use of immunosuppressive therapies for organ transplantation as well as the use of broad spectrum antibiotic therapy [29]. This study determined the prevalence and characteristics of genitourinary candidiasis as well as the species of* Candida* among attendees of Sexually Transmitted Disease (STD) clinic of Ladoke Akintola University Teaching Hospital, Osogbo, Nigeria. The prevalence of 47.4% reported in our study agrees with rates reported in previous studies of Enweani et al. [31], Sehgal [32], and Okungbowa et al. [33] conducted on similar populations and who recorded 40.7%, 54%, and 57.3% rates, respectively. However, the rate in our study was far less than the rate reported in a more recent study by Farooqi et al. [34]. The differences in rates reported may be attributed to the geographical variability, period of specimen collection, methods of identification, and differences in study population.

*Candida glabrata,* a non-*C. albicans* Candida, was the most prevalent causative agent of genitourinary candidiasis in this study with 46.9%. This is in conformity with Okungbowa et al. [33] who reported* C. glabrata* as the most prevalent* Candida* species in their study with 33.7% and Srujana et al. [35] with 50.4%. Other studies that have reported non-*C. albicans* as predominant isolates such as Malini [36] with 54.1% and Ragini et al. [37] with 69.7% also agree with the 66.3% of non-*C. albicans* Candida isolates reported in our current study. However some recent studies [6, 38] still reported* C. albicans* as the predominant* Candida* species isolated in genitourinary candidiasis. The significance of our finding is in the light of postulates by some investigators that* C. glabrata* may have emerged as breakthrough vaginal infections in women receiving long-term maintenance low-dose fluconazole prophylaxis [24]. There is a high possibility in our environment that* C. glabrata* and other non-*C. albicans* may have been responsible for recurrent vaginal candidiasis and apparent failed treatment seen in many women treated with ketoconazole and fluconazole, two most important antifungi azoles routinely used in our hospital for treating candidiasis (personal communication). We also noted higher* Candida* recovery from urine specimen, especially for the non-*C. albicans* Candida, than from vulvovaginal or urethral swabs. Examination of urine may therefore serve as credible alternative, especially when vulvovaginal swab cultures are persistently negative, but the patients continue to experience symptoms of genitourinary candidiasis.

In this study, genitourinary candidiasis occurred most frequently in the age groups 31–45 and 16–30 years which together had prevalence rate of 40.1%. This age bracket, which encompasses teenagers, adolescents, and young adults, represents the most sexually active population group, with predisposition to increased sexual activities, an important risk factor for genitourinary candidiasis [5, 16, 22, 39]. This observation has been similarly reported by other researchers in Nigeria [33, 38, 40] and elsewhere [32]. It is however interesting to note that we isolated* Candida* from urine of 7 out of the 10 children (age group 1–15 years) enrolled in the study, 6 of whom were prepubertal girls and 1 was a male child. All 7 had symptoms of urinary tract infection and non-*C. albicans* were recovered from 5 of them.* Candida* is a normal flora of the vagina of prepubertal girls and usually kept at bay by the lactobacillus flora. However, a distortion in the normal lactobacilli balance, especially following broad spectrum antibiotic therapy, sexual activity, or use of contraceptives, may enable* Candida,* especially* C. glabrata,* to proliferate to produce symptomatic genitourinary disease [41–43]. We could not ascertain the factors that predisposed these children to candidiasis. However, three of them had received antibiotic therapy two weeks before the study. The preponderance of females attending our STD clinic and the high prevalence of genitourinary candidiasis in them have previously been established in our institution [44].

Genitourinary candidiasis can be symptomatic or asymptomatic [23, 24]. In our study, only 41.1% of the clinic attendees were symptomatic of candidiasis, most of whom were pregnant women and diabetic patients, in agreement with what has been previously reported [19, 45]. The apparently healthy attendees (who were probably venereophobic) and the HIV-infected patients were largely asymptomatic, although* Candida* was isolated from their urine or vulvovaginal swabs. One limitation in our study was that we did not perform quantitative* Candida* culture to determine whether density of organisms correlated with symptomatology; hence we are unable to provide explanation why these groups of attendees were asymptomatic. However, it is generally established that high inoculum of fungi organisms correlate with fungi pathogenicity and symptomatology [3, 46]. Asymptomatic genitourinary candidiasis usually does not require specific treatment as correcting any risk factor can result in remission of disease. However, immunocompromised patients, who are asymptomatic carrier of* Candida,* are at risk of developing invasive fungal infections; hence all HIV-infected and diabetes mellitus patients in our study and all symptomatic patients were offered appropriate antifungi therapy.

## 5. Conclusion

The findings in this study showed that* C. albicans*, usually reported to be the most frequently isolated species in genitourinary candidiasis, was not the main species recovered. The non-*C. albicans* Candida, especially* C. glabrata,* were most frequently isolated. Phenotypic methods of identification used in this study proved adequate for the primary isolation, presumptive identification, and speciation of* Candida.*

## Figures and Tables

**Table 1:** Sociodemographic characteristics of Sexually Transmitted Disease (STD) clinic attendees in LAUTECH Teaching Hospital, Osogbo, Nigeria.

Characteristics	Number of attendees			%
(1) Age group (years)	Male	Female	Total	
1–15	2	8	10	2.7
16–30	20	84	104	28.2
31–45	51	148	199	53.9
45–60	8	35	43	11.6
>60	10	3	13	3.5
Total	**91**	**278**	**369**	**100**
(2) Marital status				
Married	142			38.5
Married but separated	58			15.7
Married but divorced/widowed	75			20.3
Single	94			25.5
(3) Educational status				
Tertiary	152			41.2
Primary/secondary	125			33.9
Artisan/skilled workers/uneducated	92			24.9
(4) Occupational status				
Full-time employment	162			43.9
Part-time employment	114			30.9
Not employed	93			25.2
(5) Categories of attendees				
Pregnant women	86			23.3
HIV-infected	94			25.5
DM	70			18.9
UTI	33			8.9
Apparently healthy	86			23.3

**Table 2:** Distribution of genitourinary candidiasis by age group and gender among STD clinic attendees of LAUTECH Teaching Hospital, Osogbo, Nigeria.

Age group (years)	Gender		Total	Prevalence rate (%)	95% CI	OR	*p* value
	Male	Female					
1–15 16–30 31–45 46–60 >60	1 5 6 1 5	6 57 80 12 2	7 62 86 13 7	1.9 16.8 23.3 3.5 1.9			*p* = 0.0034

Total	18	157	175	47.4	0.1077–0.3353	0.1900	*p* < 0.0001

**Table 3:** Symptomatology of genitourinary candidiasis among STD clinic attendees in LAUTECH Teaching Hospital, Osogbo, Nigeria.

Categories	Number of attendees	Number of those positive for *Candida *	Number of symptomatic attendees	Number of asymptomatic attendees
Pregnancy	86	51	39	12
HIV-infected	94	40	1	39
DM	70	34	25	9
UTI	33	10	7	3
Apparently healthy	86	40	0	40

Total	369	175	72	103

**Table 4:** Frequency of occurrence of *Candida* species among STD clinic attendees of LAUTECH Teaching Hospital, Osogbo, Nigeria.

*Candida* species	Frequency	Percentage (%)
*Candida glabrata *	82	46.9
*Candida albicans *	59	33.7
*Candida dubliniensis *	17	9.7
*Candida tropicalis *	10	5.7
*Candida krusei *	3	1.7
*Candida lusitaniae *	3	1.7
*Candida utilis *	1	0.6

Total	175	100

**Table 5:** Distribution of *Candida albicans* and non-*C. albicans* Candida species among STD clinic attendees of LAUTECH Teaching Hospital, Osogbo, Nigeria.

Categories of attendees	*C. albicans* (%)	Non-*C. albicans* Candida (%)	Total (%)	*X* ^2^	*p* value
Pregnancy	17	34	51	2.495	*p* = 0.6455
HIV-infected	14	26	40		
DM	8	26	34		
UTI	4	6	10		
Apparently healthy	16	24	40		

Total	59 (33.7)	116 (66.3)	175 (100)		

**Table 6:** Recovery rate of *Candida* species from clinical specimens obtained from STD clinic attendees of LAUTECH Teaching Hospital, Osogbo, Nigeria.

Clinical specimens	Number of samples	Number of those positive for *Candida *	% Recovery rate	*X* ^2^	*p* value
Vulvovaginal swab	266	124	46.6	27.678	*p* < 0.0001
Urine	78	50	64.1		
Urethral swab	25	1	4.0		

Total	369	175	47.4		
